# Unforeseen and Immediate: A Case of a Post-Watchman Device-Related Thrombus

**DOI:** 10.7759/cureus.63442

**Published:** 2024-06-29

**Authors:** Samdish Sethi, Anton Stolear, Maxim Dulgher, Nneka Nwokeocha, Victor Mejia, Evgeny Shkolnik

**Affiliations:** 1 Cardiology, Yale University/Bridgeport Hospital, Bridgeport, USA; 2 Internal Medicine, Nuvance Health, Norwalk Hospital, Norwalk, USA; 3 Cardiology, Yale School of Medicine, New Haven, USA

**Keywords:** anticoagulation, atrial fibrillation, left atrial appendage occlusion, watchman, device-related thrombus

## Abstract

Atrial fibrillation (AFib) is recognized as a risk factor linked to arterial thromboembolism stemming from blood clot formation in the left atrium, associated with increased morbidity and mortality. Most of these thrombi originate in the left atrial appendage (LAA). Oral anticoagulation (OAC) therapy can help mitigate this risk. LAA occlusion (LAAO) has emerged as an option for patients who cannot safely tolerate long-term OAC. Watchman is one of the commonly used devices with a favorable safety profile demonstrated in numerous studies. One of the most concerning complications of LAAO is device-related thrombus (DRT), which may form on the atrial side of the device and potentially lead to embolization. We present a rare case of immediate DRT formation following the deployment of a Watchman device in a 78-year-old male with persistent AFib. Despite appropriate periprocedural management, a thrombus was observed immediately post implantation. This case emphasizes the need for vigilant surveillance, prompt diagnosis, and therapeutic intervention to manage such complications. The patient was successfully managed with a heparin drip, leading to thrombus resolution. This report underscores the complexities of managing DRT and the importance of ongoing research to optimize outcomes for patients undergoing LAAO.

## Introduction

Atrial fibrillation (AFib) is a significant risk factor for arterial thromboembolism, largely due to the formation of blood clots within the left atrium [1]. These thrombi predominantly originate in the left atrial appendage (LAA), a small, ear-shaped sac in the muscle wall of the left atrium. Once formed, these clots can dislodge and travel through the bloodstream, potentially causing serious complications such as stroke or systemic arterial embolism, the risk of which can be significantly reduced with oral anticoagulation (OAC) therapy. However, for those who are unable to safely tolerate long-term OAC, LAAO has become a viable alternative, which can be complicated by device-related thrombus (DRT) in up to 3.8% of cases [2].

We present a rare case of immediate DRT formation following the deployment of a Watchman device, highlighting the complexities of managing such complications. By presenting this case, we seek to highlight the complexities and challenges associated with managing immediate DRT in patients undergoing LAAO, emphasizing the importance of vigilant surveillance, prompt diagnosis, and appropriate therapeutic interventions to optimize patient outcomes.

## Case presentation

A 78-year-old male, with persistent AFib with a CHA2DS2-VASc score of 6 and a history of ischemic stroke, was referred for elective LAAO with a Watchman device given the inability to safely tolerate long-term OAC due to recurrent gastrointestinal bleeding and hematuria.

The patient has been taking OAC (apixaban) uninterruptedly for several years until the day of the procedure. He had no personal or family history of known clotting disorders. Periprocedural transesophageal echocardiogram (TEE) demonstrated normal biventricular function and no evidence of thrombus in the left atrium or LAA. Standard measurements of the LAA orifice were conducted. Intravenous heparin was administered before transseptal puncture, and activated clotting time was maintained in the therapeutic range throughout the procedure. A transseptal puncture was performed, and a 35 mm Watchman device was delivered under fluoroscopic and TEE guidance. The PASS (proper position, anchoring, size, and seal) criteria were confirmed before the release of the device.

**Figure 1:**
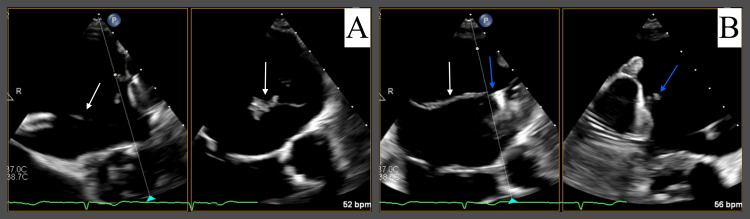
Mobile linear thrombus measuring up to 3.5 cm (white arrow) was observed at a core wire attachment site of the Watchman device (blue arrow) visualized on TEE. Mid-esophageal biplane views at 0° (A) and 80° (B) focusing on the mobile thrombus.

**Figure 2:**
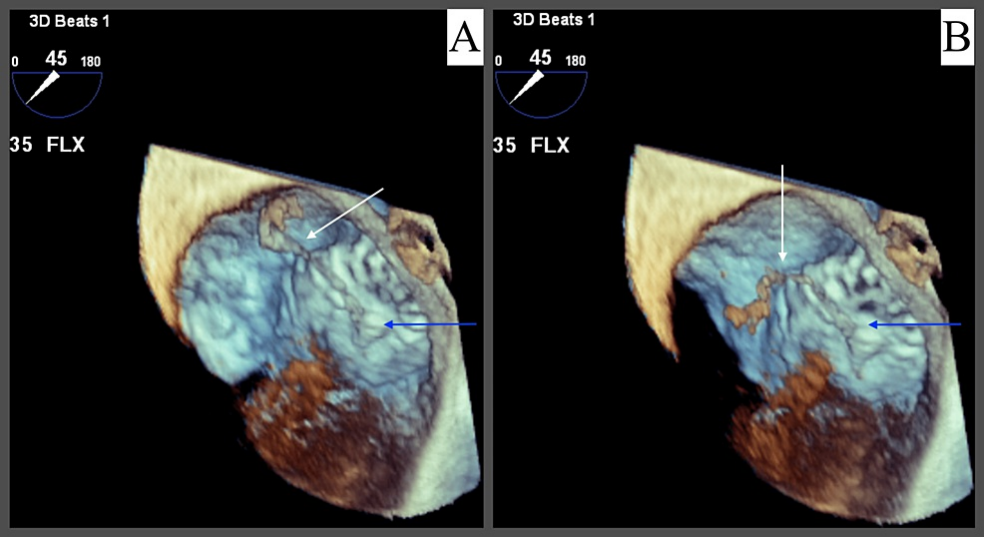
3D TEE (A and B) of mobile thrombus (white arrow) attached to the Watchman device (blue arrow).

Immediately following the device release, a mobile linear structure measuring up to 3.5 cm, consistent with thrombus, was observed at a core wire attachment site of the Watchman device (Figures 1-2), despite activated clotting time being within the therapeutic/supratherapeutic range (from 318 to > 400 seconds) throughout the entire procedure. Attempt to aspirate thrombus through the Watchman sheath was unsuccessful.

The patient was admitted to the intensive care unit for close monitoring of hemodynamics and frequent neurological assessments. Therapeutic anticoagulation with heparin infusion was maintained, and a consultation with cardiothoracic surgery was requested. Surgical intervention for clot removal was considered if anticoagulation therapy proved ineffective in resolving the thrombus. The neurologic exam remained stable with no evidence of neurological deficits.

**Figure 3:**
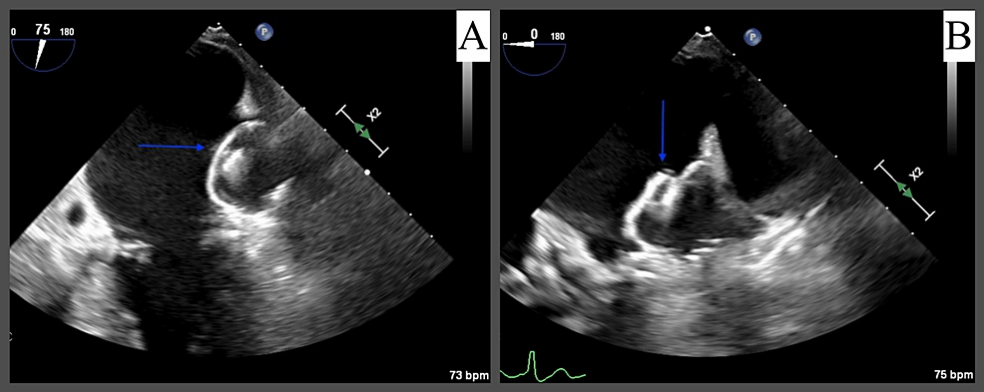
Resolution of a previously seen mobile thrombus on follow-up TEE three days later. Watchman device indicated by the blue arrow. Mid-esophageal views at 75° (A) and 0° (B) focusing on the Watchman device at the attachment site of the previously seen thrombus.

A follow-up TEE conducted two days post-procedure demonstrated a well-positioned Watchman device, with complete resolution of the thrombus (Figure 3).

The patient was transitioned from a heparin infusion to apixaban and discharged home in stable condition. Follow-up TEE six weeks after the procedure confirmed a well-seated Watchman device, trivial peri-device leak, and no evidence of thrombus on the atrial side of the device.

## Discussion

LAAO devices are commonly used in patients with AFib who are not suitable for long-term anticoagulation therapy. However, due to the foreign body nature of these intravascular devices, they can precipitate DRT, posing a potential risk of subsequent embolization before the endothelialization process is completed.

Common practice involves conducting a follow-up TEE approximately six weeks post-implantation of the Watchman device to verify proper device positioning, evaluate for residual leak, and assess for DRT. However, data from PROTECT AF (Watchman Left Atrial Appendage System for Embolic Protection in Patients with Atrial Fibrillation) and PREVAIL (Prospective randomized evaluation of the Watchman Left Atrial Appendage Closure device in patients with atrial fibrillation versus long-term warfarin therapy) trials revealed that a significant proportion of DRTs were detected beyond the one-year mark [1]. In a meta-analysis comprising 10,154 patients across 66 studies who underwent post-LAAO surveillance imaging, the pooled incidence of DRT was found to be 3.8% and associated with increased risk of ischemic events [2]; however, immediate clot formation after the Watchman device deployment is an exceedingly rare occurrence, with only several cases documented thus far [3].

On the contrary findings from a prospective real-world registry of the device (EWO-LUTION (Registry on Watchman Outcomes in Real-Life Utilization)) revealed no notable disparities in the annual rates of stroke, transient ischemic attack, or systemic embolization in patients with or without DRT (1.7% vs 2.2% per year, respectively) [4]. However, it is essential to interpret these findings cautiously, as the lack of DRT at the time of adverse ischemic events does not rule out its presence just before the event.

Identifying predictors of DRT has been challenging due to its low incidence and variability in surveillance imaging. Patient-related risk factors include female sex, prior cerebrovascular accident or transient ischemic attack, permanent AFib, LAA anatomy, reduced LVEF, chronic kidney disease, genetic resistance to antiplatelet therapy, and hypercoagulability from systemic illness. Procedural factors, such as the operator’s experience, implantation depth, and device type and size, also play a role [1,5,6]. In our presented case, patient-related risk factors included a history of prior stroke and permanent AFib.

In patients with multiple risk factors for DRT, frequent surveillance, especially during the first year post-LAAO, may be considered to facilitate early detection and prompt treatment. Resuming anticoagulation upon detection of DRT may help mitigate the risk of subsequent ischemic stroke [1]. However, uncertainty remains regarding the optimal treatment regimen and its duration.

Anticoagulation therapy is the standard initial treatment for device-related thrombus. However, complications such as bleeding and non-responsiveness to anticoagulation therapy have been reported. Consequently, alternative treatments, including surgical intervention, were attempted for patients who do not respond to anticoagulation therapy or have large, mobile DRT. One study reported DRT removal and covering the orifice of the left atrial appendage with an autologous pericardial patch [7]. Recently, percutaneous thrombectomy using a Penumbra Lightning catheter with a simultaneous cerebral embolic protection device (Sentinel) has been described as a treatment modality for patients with DRT [8]. In our case, conservative management with a heparin infusion successfully resolved the DRT, consistent with findings from previously reported meta-analyses [9].

## Conclusions

LAAO has become a well-established alternative for stroke prevention in patients with AFib who have contraindications to long-term OAC. This case highlights the complexities and periprocedural challenges associated with DRT immediately following LAAO with the Watchman device. The immediate appearance of DRT underscores the need for careful evaluation and highlights the uncertainty regarding the optimal post-procedural antithrombotic regimen. The unpredictable timing and patterns of DRT present significant clinical challenges. There is no consensus on the best strategy to prevent DRT following LAAO. To address these challenges, prospective registries and studies are needed to identify predisposing factors for DRT and to understand its clinical significance. Research efforts should focus on determining the impact of DRT on long-term outcomes and developing targeted prevention strategies. Advancing our knowledge in these areas will enhance patient safety and improve the overall success of LAAO as a stroke prevention strategy for AFib patients who cannot tolerate long-term OAC therapy.
